# Evaluating the safety of anti‐amyloid monoclonal antibody infusions in alternative sites of care

**DOI:** 10.1002/alz.084237

**Published:** 2025-01-09

**Authors:** Christine Miller, Don Filibeck, Lauren Pica, Barbara Prosser

**Affiliations:** ^1^ Soleo Health, Frisco, TX USA

## Abstract

**Background:**

The approval of anti‐amyloid monoclonal antibodies (mAbs) provides a novel approach to the treatment of Alzheimer’s disease. Infusions in alternative sites of care can benefit the patient financially and logistically, but coverage is largely payor dependent. The purpose of this study is to describe observations from this national complex specialty pharmacy around the safety of anti‐amyloid mAb infusions in alternative sites of care, including the home.

**Method:**

A multidisciplinary team conducted a retrospective review of medical records for patients receiving anti‐amyloid mAbs from 01/01/2023‐12/31/2023, including infusion‐related reactions and adverse events reported by the patient or caregiver. Thirty patients received a total of 194 infusions administered by registered nurses employed by this organization. Fifty‐seven infusions were provided in the home environment, and 137 infusions were conducted in the pharmacy’s ambulatory infusion centers.

**Result:**

Infusion‐related reactions occurred in 14.5% of infusions: hypotension 11%, hypertension 1.5%, headache 1.5%, and paresthesia 0.5%. The infusion rate was slowed during 1% of infusions. No infusions were discontinued due to infusion‐related reactions. No signs or symptoms of hypersensitivity or other infusion‐related reactions were observed by the nurse or reported by the patient during drug administration or the post‐infusion observation period. Patients completed mini‐mental state exams with the registered nurse during 94% of clinical assessments performed with the infusions. Symptoms associated with amyloid‐related imaging abnormalities (ARIA) were reported during 18% of assessments at the following rates: confusion 11%, headache 7%, altered mental status 6%, disorientation 3%, dizziness/vertigo 3%, irregular verbal communication 2%, gait difficulty 1%, visual disturbance 1%. One patient discontinued therapy after confirmatory MRI. Other adverse events reported during ≥2% of infusions include falls 7%, enlarged vocal cords 4%, nausea 2%, and flu‐like symptoms 2%.

**Conclusion:**

With the prevalence of Alzheimer’s disease expecting to reach 13 million by 2050, a wider scope of services needs to be considered for this patient population. Anti‐amyloid mAb infusions can be safely and successfully administered in alternative sites of care, including the home.

